# A comparative study of adult and adolescent maternal care continuum following community-oriented interventions in Cambodia, Guatemala, Kenya, and Zambia

**DOI:** 10.1371/journal.pone.0261161

**Published:** 2022-01-13

**Authors:** Anbrasi Edward, Younghee Jung, Grace Ettyang, Chhea Chhorvann, Casey Risko, Annette E. Ghee, Jane Chege

**Affiliations:** 1 Department of International Health, Johns Hopkins Bloomberg School of Public Health, Baltimore, Maryland, United States of America; 2 Neglected Tropical Disease, World Health Organization, Dili, Timor-Leste; 3 School of Public Health, College of Health Sciences, Moi University, Kesses, Kenya; 4 National Institute of Public Health, Phnom Penh, Cambodia; 5 Department of Global Health, University of Washington, Seattle, Washington, United States of America; 6 World Vision International, Washington, DC, United States of America; University of Botswana, BOTSWANA

## Abstract

**Background:**

The coverage for reproductive care continuum is a growing concern for communities in low- income economies. Adolescents (15–19 years) are often at higher odds of maternal morbidity and mortality due to other underlying factors including biological immaturity, social, and economic differences. The aim of the study was to examine a) differences in care-seeking and continuum of care (4 antenatal care (ANC4+), skilled birth attendance (SBA) and postnatal care (PNC) within 24h) between adult (20–49 Years) and adolescents and b) the effect of multilevel community-oriented interventions on adolescent and adult reproductive care-seeking in Cambodia, Guatemala, Kenya, and Zambia using a quasi-experimental study design.

**Methods:**

In each country, communities in two districts/sub-districts received timed community health worker (CHW) household health promotion and social accountability interventions with community scorecards. Two matched districts/sub-districts were selected for comparison and received routine healthcare services.

**Results:**

Results from the final evaluation showed that there were no significant differences in the care continuum for adolescents and adults except for Kenya (26.1% vs 18.8%, p<0.05). SBA was significantly higher for adolescents compared to adult women for Guatemala (64% vs 55.5%, p<0.05). Adolescents in the intervention sites showed significantly higher ANC utilization for Kenya (95.3% vs 84.8%, p<0.01) and Zambia (87% vs 72.7%, p<0.05), ANC4 for Cambodia (83.7% vs 43.2%, p<0.001) and Kenya (65.9% vs 48.1%, p<0.05), SBA for Cambodia (100% vs 88.9%, p<0.05), early PNC for Cambodia (91.8% vs 72.8%, p<0.01) and Zambia (56.5% vs 16.9%, p<0.001) compared to the comparison sites. However, the findings from Guatemala illustrated significantly lower care continuum for intervention sites (aOR:0.34, 95% CI 0.28–0.42, p<0.001). The study provides some evidence on the potential of multilevel community-oriented interventions to improve adolescent healthcare seeking in rural contexts. The predictors of care continuum varied across countries, indicating the importance of contextual factors in designing interventions.

## Introduction

Pregnancy-complications and childbirth are the leading causes of death among adolescent girls, especially in low-and-middle-income economies where 95% of the world’s 16 million adolescent births occur every year [1, 2]. The failure to specifically address this age cohort in the Millennium Development Goal efforts, prompted the call for increased attention in the Sustainability Development Goals (SDGs) [3, 4].

The continuum of care for maternal, newborn, and child health has been recognized as a key program strategy to address maternal, neonatal, and child mortality [5]. Several studies have illustrated the association of antenatal care (ANC) with facility-based delivery [6–8]. To effectively address maternal and neonatal mortality, skilled birth attendance (SBA) and access to facilities that provide quality obstetric and newborn care is essential, yet 70% of births among women belonging to the lowest wealth quintiles in Sub-Saharan Africa, South East Asia, and South Asia occur at home [9].

A myriad of socioeconomic and environmental factors influences reproductive care-seeking practices in rural areas [6, 10–13]. These range from geographical and financial access barriers, maternal education and employment, marital status, parity, wealth status, transportation, decision dynamics, and cultural practices. A recent review showed strong evidence that household level community health worker (CHW) health promotion can effectively reduce delays in maternal care-seeking and promote SBA delivery [14]. Social accountability mechanisms that integrate local governance and accountability measures to foster community and facility responsiveness have also shown to improve maternal care-seeking practices [15]. If effectively implemented, these community-oriented interventions, have the potential to address local barriers and optimize care-seeking.

Few studies have examined the effect of these community-oriented interventions on adolescent reproductive care-seeking. A multilevel analysis from 21 Sub-Saharan African countries showed that teenagers have poorer utilization of maternal health care, including inadequate ANC and SBA for deliveries than older women, after controlling for parity, educational status, premarital births, and urban/rural residence [16]. Other studies reported that younger women are likely to follow modern methods and, therefore, have a stronger preference for facility deliveries [6, 17, 18].

This study was designed as part of a larger multi-country research in Cambodia, Guatemala, Kenya, and Zambia, to determine the impact of community-oriented interventions on maternal and child health. The research was a collaborative effort between Johns Hopkins University, the National Institute of Public Health in Cambodia, Institute of Nutrition of Central America and Panama in Guatemala, Moi University School of Public Health in Kenya, and the Institute of Economic and Social Research at the University of Zambia. Results from the larger research study, including SBA, have been published earlier [19–21]. Timely CHW household health promotion complimented by social accountability mechanisms showed significantly higher odds for SBA (aOR = 7.48; CI: 3.87,14.5) for Cambodia, controlled for other factors [19]. Type of CHW services; inclusion of family members in pregnancy-related discussions, CHW follow up after referral, showed significantly higher odds of SBA for some countries. No new or additional data was obtained, or additional experiments performed in this study, except for the statistical analysis for determining differences adolescent and adult care seeking and care continuum. The larger study focused on several other elements of the integrated intervention package including handwashing practices, illness care-seeking for children, nutrition outcomes, quality of care for IMCI in health facilities etc.

This study examines the differences in reproductive care-seeking between adolescent and adult women and the effect of community-oriented interventions on care-seeking in both age cohorts.

## Methods

The study was part of a larger research trial that was conducted in Cambodia, Guatemala, Kenya, and Zambia. Ethical clearance was obtained from the Johns Hopkins Bloomberg School of Public Health Institutional Review Board (IRB # 00004986), and the Institutional Review Boards of the local research institutions (National Institute of Public Health in Cambodia, Institute of Nutrition of Central America and Panama in Guatemala, Moi University School of Public Health in Kenya, and the Institute for Economic and Social Research at the University of Zambia. Based on the country ethical protocols, written informed consent in the local language was obtained from all study participants in Kenya (Swahili), Zambia (Tonga, Nyanja, Lozi), and Guatemala (Spanish) and verbal informed consent was obtained from all study participants in Cambodia (Khmer), prior to administering the surveys and privacy and confidentiality was ensured. The consent procedures were approved by the institutional review boards in all countries and the Johns Hopkins Bloomberg School of Public Health.

In each country, four districts/sub-districts were selected from the World Vision Area Development Programs, which integrate a range of services including health, nutrition, water and sanitation, food security, education, and child protection. Two districts/sub-districts in each country were purposively assigned to the intervention arm (Table 1), which included multilevel intervention strategies: 1) household-level CHW health promotion, which entailed timed visits during specific stages of pregnancy and early childhood complimented by 2) community and facility level social accountability interventions using community scorecards to strengthen community-facility linkages, enhance community knowledge of facility entitlements, and support facility performance [22]. A previously published study provides a description of the CHW services in each country context [19].

**Table 1:** Selected study sites in each country—Districts/sub districts.

Study Arm	Cambodia	Guatemala	Kenya	Zambia
Intervention	Chulkiri	Comapa	Karemo	Luampa
Intervention	Pouk	Nuevo Amanecer	Katito	Magoye
Comparison	Prasath Balang	Apas	Kegonga-Ntimaru	Choongo
Comparison	Tbeng Meanchey	Tinamit Junam	Magunga	Nyimba

Two matched districts or sub-districts were assigned to the comparison arm of the study and received routine district health services, including CHW services and interventions from World Vision and other non-governmental organizations. Matching of the intervention and comparisons sites was based on sociodemographic characteristics and other factors, including population size, migratory patterns, health facility access, disease burden, presence of other health and non-health development programs, and maturity and capacity of the World Vision Area Development programs. In all study sites, joint facility and community health management councils were established or enhanced through training and supportive supervision based on the Global Fund’s Community Systems Strengthening Framework [23].

Sample size was estimated to detect a significant difference in differences in SBA with an effect size of 15%. Required sample size was based on a two-sided α = .05, power, (1-*β*) = 0.80. A 5% non-response and 5% lost to follow up rate, and design effect of 1.2 was factored to determine the final sample size for women recently delivered. We used a modified care continuum construct with the following elements: 4 or more ANC visits (defined as pregnancy-related care at a health facility or hospital) + SBA (assisted delivery by a doctor, nurse, or midwife) and early PNC (Post Natal Care in a facility or hospital within 24 hours of delivery) based on previous studies [24, 25].

Enumeration teams from each site performed a household listing to create sampling units. In sites, where an updated household listing was available from the central statistics office, a preliminary survey was used to update this listing. The sample was selected from each sampling unit proportionate to the population size. For Zambia, we used the DHS Standard Enumeration Areas. Prior to the household survey, eligible households (i.e., a female resident who had delivered in the past two years, was currently pregnant, or had a child under five years) were identified. Households were randomly selected from this listing to obtain the required sample from that sampling unit. All eligible households were included if the estimated sample size was not achieved. If more than one woman per household was eligible, one woman was randomly selected.

The survey procedures and instruments used by the Demographic Health Surveys were modified for the study [26]. Data on socio-demographic characteristics, food security, and household assets were obtained by interviews with heads of households. Data on reproductive health and care-seeking was obtained from women, aged 15–49 years, who had delivered in the last two years. All women who reported a live birth were included in this analysis. Trained survey teams conducted the interviews and informed consent was obtained from all participants. Standard procedures for survey administration, data quality control, storage, and security, were followed throughout the study period.

### Data analysis

We employed standard data management procedures to clean, verify and analyze the data using STATA 14 [27]. First, we performed a descriptive analysis to determine differences between adolescent and adult women. We then disaggregated the analysis by intervention and comparison sites by country. All analysis was performed by country, as contextual factors differed. A difference in difference analysis was not performed due to minor variations in the baseline instruments, data from the final evaluation is presented in this study.

Bivariate and multivariate logistic regression models were created to determine the association between care-seeking and known predictors including maternal age, education, parity, marital status, wealth status, and treatment arm of the study. Collinearity was tested for all the independent variables. Cases with missing data for the independent variables were excluded in the regression models. Wealth quintiles were constructed employing a principal component analysis with a combination of household assets (television, radio, bicycle, etc.) and household type (roofing, drinking water source, and sanitation). The construct for the wealth quintile was based on previous studies and differed from that used by the Demographic Health Survey [21].

## Results

### Background characteristics by country

Select background characteristics of adolescent and adult women are described in Table 2. A significantly higher proportion of adult women in Kenya resided in male-headed households compared to adolescents (88.5% vs 79.0%, p<0.001). A higher proportion of adult women were married compared to adolescent women for Guatemala (34.1% vs 61.7%, p<0.001), Kenya (59.9% vs 89.8%, p<0.001), and Zambia (50.3% vs 75.0%, p<0.001). Secondary or higher levels of education were significantly higher for adolescents than adult women for Cambodia (46.9% vs 29.9%, p<0.001), Guatemala (26.8% vs 13.1%, p<0.001), and Zambia (47.9% vs 35.4%, p<0.05).

**Table 2:** Demographic profile of study population.

Characteristics	Cambodia N = 2,995		Guatemala N = 1,992		Kenya N = 2,581		Zambia N = 1,057	
	Adolescent	Adult	Adolescent	Adult	Adolescent	Adult	Adolescent	Adult
	N (% or mean)	N (% or mean)	N (% or mean)	N (% or mean)	N (% or mean)	N (% or mean)	N (% or mean)	N (% or mean)
	130	2,865	164	1,828	208	2,373	169	888
**Male Headed Households**	114 (87.7)	2,564 (89.7)	147 (89.6)	1,680 (91.9)	162 (79.0)	2,080 (88.5)***	85 (50.6)	494 (56.7)
**Mean Family Size**	130 (5.1)	2,865 (4.9)	164 (3.5)	1,828 (5.1)***	208 (4.2)	2,371 (4.9)***	169 (4.6)	885 (5.4)***
**Mother Marital status**								
Married	126 (96.9)	2,815 (98.3)	56 (34.1)	1,126 (61.7)***	124 (59.9)	2,123 (89.8)***	85 (50.3)	664 (75.0)***
Single/Divorced/Widow	4 (3.1)	50 (1.7)	108 (65.9)***	700 (38.3)	83 (40.1)***	241 (10.2)	84 (49.7)***	221 (25.0)
**Mother’s Parity**								
First Parity	122 (93.8)***	1,054 (36.8)	-	-	16 (7.7)*	96 (4.0)*	67 (39.9)***	84 (9.5)***
Two or more	8 (6.2)	1,811 (63.2)***	164 (100.0)	1,828 (100.0)	192 (92.3)*	2,276 (96.0)*	101 (60.1)***	804 (90.5)***
**Mother’s education**								
None	8 (6.3)	619 (21.8)***	11 (6.7)	538 (29.8)***	5 (2.8)	61 (2.8)	7 (5.0)	94 (12.9)**
Primary	60 (46.9)	1,366 (48.2)	109 (66.5)*	1,030 (57.1)	137 (76.5)	1,508 (69.9)	66 (47.1)	376 (51.6)
Secondary or more	60 (46.9)***	848 (29.9)	44 (26.8)***	236 (13.1)	37 (20.7)	588 (27.3)	67 (47.9)**	258 (35.4)
**CHW visit (last pregnancy)**	57 (43.8)	1,438 (50.8)	8 (4.9)	76 (4.2)	75 (36.8)	1,081 (46.4)**	28 (16.6)	173 (19.5)
3 or more CHW visits	22 (38.6)	582 (41.0)	4 (50.0)	28 (38.4)	43 (58.1)	634 (63.5)	14 (53.8)	84 (50.9)
**Wealth Quintile**								
1^st^	40 (30.8)	779 (27.2)	41 (25.0)	427 (23.4)	43 (20.7)	447 (18.8)	38 (22.5)	200 (22.5)
2^nd^	17 (13.1)	488 (17.0)	37 (22.6)	387 (21.2)	56 (26.9)	502 (21.2)	36 (21.3)	152 (17.1)
3^rd^	29 (22.3)	592 (20.7)	38 (23.2)	339 (18.5)	41 (19.7)	427 (18.0)	33 (19.5)	180 (20.3)
4^th^	26 (20.0)	556 (19.4)	22 (13.4)	385 (21.1)*	44 (21.2)	448 (18.9)	38 (22.5)	222 (25.0)
5^th^	18 (13.8)	450 (15.7)	26 (15.9)	290 (15.9)	24 (11.5)	549 (23.1)***	24 (14.2)	134 (15.1)
**Self-decisions about household purchase**	63 (48.5)	1,875 (65.7) ***	82 (50.0)	1,221 (66.8) ***	79 (39.5)	1,375 (59.3) ***	78 (46.2)	517 (59.7) ***
**Self-decisions with or without partners on healthcare**	78 (60.0)	1,931 (67.5)	79 (48.2)	986 (53.9)	128 (63.1)	1,765 (75.9) ***	120 (71.4)	733 (84.1) ***
**Access to mobile phone**	110 (84.6)*	2,199 (76.8)	128 (78.0)	1,422 (77.8)	168 (80.8)	2,028 (85.5)	117 (69.2)	613 (69.0)

*p<0.05,

**p<0.01,

***p<0.001

t tests between adolescents and adults for each variable, by country.

No significant differences were evident for CHW visits between adolescents and adults except for Kenya, where adult women reported significantly higher proportion of CHW visits than adolescents (46.4% vs 36.8%, p<0.01). Decision-making power for household purchases was significantly higher for adult women in all four countries compared to adolescents; however, decision-making power for healthcare was significantly higher for adult women only for Kenya and Zambia. Adolescents reported significantly higher access to mobile phone than adults for Cambodia (84.6% vs 76.8%, p<0.05).

### Care-seeking differences between adolescent and adult women

In all four countries, care-seeking for ANC, ANC4+ and mean month of first ANC was not significantly different between adolescent and adult women (Table 3). Adolescent girls from Guatemala reported higher SBA (64.0% vs 55.5%, p<0.05) and facility deliveries (63.4% vs 55.4%, p<0.05) than adult women. Reported PNC was also similar between the two age cohorts. Care continuum (ANC4+SBA+PNC), was significantly higher for adult women than adolescents for Kenya only (26.1% vs 18.8%, p<0.05).

**Table 3:** Care-seeking characteristics for ANC, delivery care and PNC: Adolescent vs adults.

Characteristics	Cambodia		Guatemala		Kenya		Zambia	
	Adolescents	Adults	Adolescents	Adult	Adolescents	Adult	Adolescents	Adult
	N (% or mean)	N (% or mean)	N (% or mean)	N (% or mean)	N (% or mean)	N (% or mean)	N (% or mean)	N (% or mean)
**Prepartum care**								
ANC utilization								
*1+ Facility ANC*	121 (100.0)	2,702 (99.9)	74 (100.0)	673 (99.6)	190 (99.0)	2,127 (97.8)	128 (90.1)	677 (90.0)
*4+ ANC visits*	73 (60.3)	1,845 (68.3)	52 (70.3)	473 (70.3)	121 (63.7)	1,433 (67.4)	87 (68.0)	428 (63.2)
**Delivery Care**								
Skilled birth attendance (SBA)	121 (93.1)	2,599 (90.7)	105 (64.0)*	1,014 (55.5)	187 (91.7)	2,142 (92.0)	161 (95.3)	838 (94.7)
Facility Delivery	121 (93.1)	2,499 (87.3)	104 (63.4)*	1013 (55.4)	178 (87.3)	2,067 (88.9)	149 (88.2)	764 (86.3)
**Post-partum Care**								
*Received PNC*	120 (93.0)	2,658 (93.3)	109 (66.5)	1,140 (62.6)	169 (82.8)	1,952 (84.1)	155 (91.7)	777 (88.7)
*Early PNC (<24hrs)*	103 (85.1)	2,400 (89.8)	84 (77.1)	894 (78.1)	69 (40.4)	883 (44.6)	65 (41.9)	306 (39.2)
*ANC4 + SBA + PNC*	67 (51.5)	1,613 (56.3)	29 (17.7)	267 (14.6)	39 (18.8)	619 (26.1)*	32 (18.9)	174 (19.6)

*p<0.05,

**p<0.01,

***p<0.001

t tests between adolescents and adults by country.

### Care-seeking patterns by intervention

In Cambodia, relative to the comparison sites, in the intervention sites, a significantly higher proportion of adolescents reported ANC4 (83.7% vs 43.2%, p<0.001), facility deliveries (100% vs 88.9%, p<0.05), SBA (100% vs 88.9%, p<0.05), early PNC (91.8% vs 72.8%, p<0.01), and care continuum (77.6% vs 35.8%, p<0.001) (Table 4). Similarly, in Kenya, adolescents in the intervention sites reported significantly higher utilization for ANC, ANC4, and ANC4+SBA. In Zambia, adolescents in the intervention sites also reported significantly higher utilization for ANC, early PNC, and care continuum. However, in Guatemala, adolescents from the comparison sites reported significantly higher utilization for SBA, facility deliveries, early PNC, and care continuum.

**Table 4:** Care-seeking characteristics of adolescents and adult women in intervention vs comparison study sites—Post intervention.

Characteristics	Cambodia		Guatemala		Kenya		Zambia	
	Intervention	Comparison	Intervention	Comparison	Intervention	Comparison	Intervention	Comparison
	N = 1,254	N = 1,741	N = 898	N = 1,094	N = 1,485	N = 1,096	N = 588	N = 469
	N(%)	N(%)	N(%)	N(%)	N(%)	N(%)	N(%)	N(%)
**ANC**								
Adult	1,174 (97.4)***	1,532 (92.3)	227 (27.4)	447 (44.7)***	1,281 (94.5)***	883 (86.8)	448 (90.3)***	264 (67.3)
Adolescent	46 (93.9)	75 (92.6)	26 (37.7)	48 (50.5)	123 (95.3)**	67 (84.8)	80 (87.0)*	56 (72.7)
**ANC 4+**								
Adult	976 (81.0)***	897 (54.0)	163 (19.7)	337 (33.7)***	920 (67.8)***	563 (55.4)	275 (55.4)	224 (57.1)
Adolescent	41 (83.7)***	35 (43.2)	21 (30.4)	33 (34.7)	85 (65.9)*	38 (48.1)	50 (54.3)	48 (62.3)
**SBA**								
Adult	1,193 (99.0)***	1,406 (84.7)	357 (43.1)	657 (65.8)***	1,240 (91.4)*	902 (88.7)	463 (93.3)	375 (95.7)
Adolescent	49 (100.0)*	72 (88.9)	33 (47.8)	72 (75.8)***	118 (91.5)	69 (87.3)	90 (97.8)	71 (92.2)
**Early PNC (<24h)**								
Adult	1,126 (93.4)***	1,288 (77.6)	215 (25.9)	684 (68.5)***	540 (39.8)*	356 (35.0)	262 (52.8)***	47 (12.0)
Adolescent	45 (91.8)**	59 (72.8)	19 (27.5)	65 (68.4)***	39 (30.2)	30 (38.0)	52 (56.5)***	13 (16.9)
**ANC4 + SBA + PNC**								
Adult	913 (75.8)***	700 (42.2)	45 (5.4)	222 (22.2)***	394 (29.1)***	225 (22.1)	146 (29.4)***	28 (7.1)
Adolescent	38 (77.6)***	29 (35.8)	4 (5.8)	25 (26.3)**	25 (19.4)	14 (17.7)	25 (27.2)**	7 (9.1)

*p<0.05,

**p<0.01,

***p<0.001

t tests between intervention and comparison sites by age group.

Likewise, adult women in the intervention sites had significantly higher ANC, ANC4, SBA, early PNC, and care continuum than women in comparison sites for Cambodia and Kenya. This difference was significant only for ANC and early PNC in Zambia. A similar pattern of higher utilization for ANC4, early PNC, and SBA was evident for adult women in the comparison sites versus the intervention sites in Guatemala.

### Determinants of care continuum

Results from the multivariate regressions showed that adult women in Cambodia had a significantly greater odds (aOR 1.60, 95% CI: 1.08–2.39, p<0.05) of achieving the care continuum, than adolescent girls (Table 5). Age difference was not significant for women in other countries for care continuum when controlled for other factors. Other significant determinants were primary or secondary education for Cambodia and Guatemala, marital status for Guatemala, parity for Cambodia, and wealth quintile for all countries. The odds of achieving the care continuum were significantly higher for women in the intervention sites, for all countries (Cambodia aOR 3.47, 95% CI: 2.9–4.1, p<0.001, Kenya aOR 1.2, 95% CI: 1.01–1.45, p<0.05, Zambia aOR 2.17, 95% CI: 1.43–3.28, p<0.001), except for Guatemala (aOR 0.34, 95% CI: 0.28–0.42, p<0.001), where women in the comparison sites showed greater odds of achieving the care continuum.

**Table 5:** Multivariate logistic regression of factors associated with care continuum (ANC4+SBA+PNC).

	*Cambodia*	*Guatemala*	*Kenya*	*Zambia*
*Care-seeking Continuum*	*OR (95%CI)*	*OR (95%CI)*	*OR (95%CI)*	*OR (95%CI)*
**Marital status** (ref, (Single/divorced/widowed)	1.000	1.000	1.000	1.000
Married	1.434 [0.789,2.606]	0.811* [0.662,0.993]	1.122 [0.852,1.477]	1.05 [0.668,1.652]
**Mother’s Education** (ref, none)	1.000	1.000	1.000	1.000
Primary	1.608*** [1.308,1.977]	1.744*** [1.395,2.179]	0.924 [0.545,1.568]	1.316 [0.712,2.431]
Secondary or more	2.094*** [1.653,2.652]	4.623*** [3.192,6.695]	1.471 [0.852,2.539]	1.31 [0.673,2.550]
**Mother’s age** (ref, <20y)	1.000	1.000	1.000	1.000
≥ 20y	1.603* [1.077,2.388]	0.911 [0.632,1.314]	1.249 [0.888,1.755]	0.994 [0.566,1.744]
**Parity** (ref, 2 or more)	1.000	1.000	1.000	1.000
1^st^ pregnancy	1.205* [1.015,1.431]		1.222 [0.811,1.840]	0.607 [0.351,1.049]
**Wealth Quintile** (ref, lowest)	1.000	1.000	1.000	1.000
Low	1.426** [1.136,1.790]	1.138 [0.859,1.507]	1.224 [0.954,1.570]	0.507* [0.261,0.986]
Middle	1.954*** [1.527,2.501]	1.164 [0.866,1.566]	1.144 [0.818,1.600]	0.532 [0.275,1.029]
High	2.175*** [1.686,2.806]	2.032*** [1.506,2.741]	1.330* [1.015,1.741]	0.658 [0.335,1.290]
Highest	2.064*** [1.574,2.706]	4.109*** [2.877,5.870]	1.183 [0.901,1.554]	0.629 [0.293,1.354]
**Health Insurance** (ref, None)	1.000	1.000	1.000	1.000
Health insurance	1.042 [0.872,1.245]			
Decisions about healthcare (ref, others)	1.000			
Self-decision with/without husbands/partners	1.118 [0.941,1.328]			
**Treatment** (Ref, Comparison)	1.000	1.000	1.000	1.000
Intervention	3.471*** [2.927,4.117]	0.341*** [0.280,0.416]	1.213* [1.014,1.452]	2.167*** [1.430,3.283]
Constant	0.115*** [0.053,0.249]	1.033 [0.670,1.594]	0.309*** [0.163,0.585]	5.616*** [2.320,13.598]
Total N	2,956	1,967	2,316	857

*p<0.05,

**p<0.01,

***p<0.001.

Determinants of individual aspects of care-seeking for ANC4, SBA and PNC are provided in the supplemental file (S1 Table). The predictors of care-seeking varied by country and included marital status, mother’s educational status, decision making power for healthcare, and wealth quintile. Significantly higher odds of care-seeking were evident only for ANC for adult women in Cambodia. Women who belonged to the intervention sites showed greater odds of ANC care-seeking in Cambodia, Kenya, and Zambia; SBA for Cambodia; and PNC for Cambodia and Zambia. The opposite was true for Guatemala, where women in the comparison site showed greater odds of care-seeking for ANC, SBA, and PNC. An interaction term for age and intervention was examined in the individual regressions for ANC, SBA and PNC, and no significance was found except for a positive direction for Kenya for PNC.

## Discussion

Maternal health care seeking is of paramount importance, as evidenced by accelerated efforts to bolster multidimensional facility and community level investments to achieve Target 3.1 of the SDGs (*By 2030*, *reduce the global maternal mortality ratio to less than 70 per 100*,*000 live births*). Increased focus on adolescents, who were ‘left behind’ in the MDG efforts, further emphasizes the need for interventions tailored for this age cohort [3].

This study showed contextual variations in maternal care-seeking characteristics between adult women and adolescents. Controlling for known confounders, Cambodian adolescents reported a significantly lower odds of ANC compared to adult women. However, adolescent in the intervention sites had significantly higher ANC4 (Cambodia and Kenya), facility deliveries and SBA (Cambodia), early PNC (Cambodia and Zambia), and care continuum (Cambodia and Zambia) than those in the control sites. In Kenya, adolescent care continuum was higher for intervention sites, but not significant. Multi-country trials have also shown that, compared to adult women of similar backgrounds, adolescents report lower utilization of maternal health services [16, 28]. A study on the DHS data from Bangladesh showed that mean age of women who reported SBA, but not ANC, was about eight years lower than the overall study participants, implying that younger women were more inclined to seek SBA for delivery [29].

Care-seeking patterns differed in Guatemala, where adolescent and adult women reported higher SBA and early PNC in the comparison sites. Qualitative findings and CHW surveys to determine implementation strength (not reported here) showed that several factors may have contributed to this finding. For example, in the intervention site Comapa, 89% of the trained CHWs reported security concerns, increased violence and crime, and reduced household visits (one per month). This may have posed critical barriers for both CHW household visits and access to facility-based care for ANC, SBA and PNC for women living in these communities. In the comparison site Apas, CHWs were trained health educators, with monetary compensation for services, which may have influenced performance.

Predictors of facility ANC (marital status, respondent education, first pregnancy, wealth quintile, healthcare decision-making, and intervention sites) and SBA (marital status, respondent education, first pregnancy, wealth quintile, and ANC4) varied by country. Aside from the variables identified in this study, other research has identified partner education, place of residence, religion, ethnicity, age at first birth, employment status, and media exposure, as key predictors of facility ANC and SBA [29, 30].

Multi-country trials have also shown that, compared to adult women of similar backgrounds, adolescents report lower utilization of maternal health services [16, 28]. A study on the DHS data from Bangladesh showed that mean age of women who reported SBA, but not ANC, was about eight years lower than the overall study participants, implying that younger women were more inclined to seek SBA for delivery [29].

Except for Guatemala, all three countries reported higher levels of care continuum for both adolescents and adults in the intervention sites, ranging from 20% (Kenya and Zambia) to 70% (Cambodia). These results are higher compared to those reported in other studies on care continuum (Ethiopia (9.1%), Ghana (8%), and Tanzania (10%)), illustrating the potential benefits of the multi-level interventions in our study [11, 13, 25].

Addressing inequities in reproductive care-seeking emerges as an important consideration for program delivery. Similar to many other studies, wealth quintile emerged as a significant predictor for ANC in Cambodia, Guatemala, and Kenya; SBA for Cambodia and Guatemala, and early PNC for Zambia [29, 31]. Analysis of data from the DHS in Kenya showed that adolescent mothers in the richest quintile were nine times more likely to use ANC and seven times more likely to have SBA than those in the poorest quintile [32]. As noted earlier, the MDGs have resulted in an unparalleled reduction in maternal and child mortality globally. However, both rates continue to increase, particularly in Asia and Africa where many women do not access ANC and SBA. Thus, global progress is eclipsing the increasing inequalities within countries that are not keeping pace with global targets. Disparities must be eliminated both across and within countries by ensuring access to critical services, especially ANC and SBA, for countries that are showing slow progress to achieve SDG targets [33].

Educational attainment also plays a key role, as relative to women with no education, those with primary education in Kenya were four times more likely to receive ANC and twice as likely to receive SBA [34]. Educational attainment emerged as key predictor for ANC (Cambodia and Guatemala) and SBA (Cambodia, Guatemala, and Kenya) in our study. Similar findings have also been observed in studies conducted in Bangladesh, Tanzania, and Nigeria [25, 29].

Decision-making power serves as another critical factor impacting adolescent care-seeking. Unmarried adolescents are unlikely to have decision making power due to their dependency on older adults in the family. Efforts for supporting adolescent healthcare decision making are additional considerations for household and community-oriented interventions. Adolescents are at higher risk for poor maternal outcomes and are at a greater risk for post-partum bleeding, anemia, and pre-eclampsia [35].

Community-based interventions mobilizing CHWs have shown to be successful in achieving universal access and quality and reducing maternal and neonatal mortality in low resource settings [36, 37]. To foster people-centered care and strengthen community governance, social accountability mechanisms have shown improvements in health service utilization and quality of care [38–41]. In the intervention sites, adolescents exposed to these interventions reported significantly higher utilization of ANC4 (Cambodia and Kenya), SBA (Cambodia), and early PNC (Cambodia and Zambia), compared to those in communities receiving standard government programming. However, Guatemalan adolescents in the comparison sites that received routine government and other program interventions reported significantly higher SBA and early PNC. As observed earlier, the security threats in one of the intervention sites in Guatemala may have been a major deterrent to care-seeking.

The findings show that multilevel programming interventions have the potential for increasing universal health coverage for the maternal care continuum and enhance adolescent care-seeking in some contexts [31]. The quasi-experimental design, low sample of adolescents, and lack of a difference in difference analysis, with baseline care-seeking data, pose considerable limitations for drawing conclusive inferences from the study. Implementation strength was also not examined to determine variations of interventions in each context. Other factors that are known predictors for SBA utilization including previous pregnancy outcomes and distance to health facilities were not factored in the regression analysis resulting in another study limitation. Reliance on respondent reports for ANC and SBA, without validation of facility records, also limits the study findings.

## Conclusion

Contextually designed multilevel interventions with social accountability mechanisms are essential to equip and engage adolescents and adult women in the care process and mitigate the barriers to universal access for reproductive health. The study provides some evidence for enhancing adolescent utilization of reproductive health services and achieve the targets for the SDGs in rural communities.

## Supporting information

S1 Table Multivariate logistic regression of factors associated with care-seeking for ANC, SBA and early PNC. (DOCX)

S1 Data (DTA)
