# Supplementary material for: A comparative study of adult and adolescent maternal care continuum following community-oriented interventions in Cambodia, Guatemala, Kenya, and Zambia
Source: PLoS One. 2022 Jan 13;17(1):e0261161. doi: 10.1371/journal.pone.0261161 (PMC8758084; doi:10.1371/journal.pone.0261161)
Supplement: S1 Table — (DOCX) [file pone.0261161.s001.docx]

A comparative study of adult and adolescent maternal care continuum following community-oriented interventions in Cambodia, Guatemala, Kenya and Zambia

Supplemental File

**S1 Table: Multivariate Logistic Regression of Factors Associated with Care-seeking for ANC, SBA and Early PNC**

|  | *Cambodia* | *Guatemala* | *Kenya* | *Zambia* |
| --- | --- | --- | --- | --- |
| ***Facility ANC*** | *OR(95%CI)* | *OR(95%CI)* | *OR(95%CI)* | *OR(95%CI)* |
| Marital status (ref, (Single/divorced/widowed) | 1.000 | 1.000 | 1.000 | 1.000 |
| Married | 2.005* [1.104,3.641] | 1.175 [0.940,1.470] | 1.449**[1.100,1.908] | 0.984 [0.719,1.347] |
| Mother’s Education (ref, none) | 1.000 | 1.000 | 1.000 | 1.000 |
| Primary | 1.399** [1.139,1.720] | 1.594***[1.212,2.097] | 0.725 [0.426,1.234] | 0.78 [0.491,1.238] |
| Secondary or more | 1.924*** [1.512,2.448] | 3.882***[2.714,5.552] | 1.023 [0.588,1.781] | 0.85 [0.518,1.396] |
| Mother’s age (ref, <20y) | 1.000 | 1.000 | 1.000 | 1.000 |
| ≥20y | 1.604* [1.068,2.407] | 0.856 [0.589,1.245] | 1.08 [0.775,1.507] | 0.98 [0.655,1.468] |
| Parity (ref, 2 or more) | 1.000 | 1.000 | 1.000 | 1.000 |
| 1^st^ pregnancy | 1.139 [0.953,1.362] |  | 1.649* [1.041,2.612] | 1.044 [0.675,1.616] |
| Health insurance (ref, none) | 1.000 |  |  |  |
| Health insurance | 0.935 [0.780,1.120] |  |  |  |
| Wealth Quintile (ref, lowest) | 1.000 | 1.000 | 1.000 | 1.000 |
| Low | 1.435** [1.147,1.795] | 0.858 [0.609,1.210] | 1.320* [1.033,1.687] | 0.946 [0.609,1.471] |
| Middle | 2.066*** [1.607,2.656] | 0.884 [0.621,1.258] | 1.388 [0.990,1.945] | 0.919 [0.604,1.400] |
| High | 2.425*** [1.860,3.161] | 1.286 [0.917,1.804] | 1.447**[1.104,1.897] | 0.934 [0.614,1.421] |
| Highest | 2.273*** [1.711,3.020] | 2.351***[1.655,3.339] | 1.659***[1.257,2.189] | 0.806 [0.489,1.329] |
| Decisions about healthcare (ref, others) | 1.000 | 1.000 | 1.000 | 1.000 |
| Self-decision with/without husbands/partners | 1.073 [0.896,1.285] | 1.351** [1.086,1.680] | 1.261* [1.028,1.546] | 0.989 [0.682,1.436] |
| Treatment (Ref, Comparison) | 1.000 | 1.000 | 1.000 | 1.000 |
| Intervention | 3.126*** [2.604,3.753] | 0.446*** 0.356,0.559] | 1.596***[1.325,1.921] | 1.377*[1.031,1.840] |
| Constant | 0.154*** [0.071,0.336] | 0.231***[0.142,0.377] | 0.682 [0.357,1.303] | 1.499 [0.768,2.928] |
| Total N | 2,934 | 1,941 | 2,253 | 846 |
|  |  |  |  |  |
| ***SBA*** |  |  |  |  |
| Marital status (ref, Single/divorced/widowed) | 1.000 | 1.000 | 1.000 | 1.000 |
| Married | 0.527 [0.152,1.825] | 0.805* [0.654,0.990] | 1.103 [0.688,1.767] | 0.712 [0.347,1.462] |
| Mother’s Education (ref, none) | 1.000 | 1.000 | 1.000 | 1.000 |
| Primary | 1.766*** [1.303,2.392] | 1.667***[1.328,2.093] | 1.738 [0.871,3.470] | 1.474 [0.623,3.488] |
| Secondary or more | 2.448*** [1.633,3.668] | 3.764***[2.573,5.504] | 5.240***[2.329,11.786] | 1.754 [0.669,4.597] |
| Mother’s age (ref, <20y) | 1.000 | 1.000 | 1.000 | 1.000 |
| ≥20y | 0.949 [0.443,2.031] | 0.913 [0.628,1.327] | 0.802 [0.445,1.445] | 0.823 [0.322,2.100] |
| Parity (ref, 2 or more) | 1.000 |  | 1.000 | 1.000 |
| 1^st^ pregnancy | 1.981*** [1.429,2.746] |  | 1.472 [0.580,3.735] | 0.915 [0.354,2.369] |
| Health insurance (ref, none) | 1.000 |  |  |  |
| Health insurance | 1.402* [1.036,1.897] |  |  |  |
| Wealth Quintile (ref, lowest) | 1.000 | 1.000 | 1.000 | 1.000 |
| Low | 1.306 [0.944,1.806] | 1.186 [0.890,1.580] | 0.789 [0.528,1.179] | 0.898 [0.361,2.231] |
| Middle | 2.107*** [1.367,3.247] | 1.185 [0.876,1.602] | 0.756 [0.438,1.306] | 1.188 [0.464,3.041] |
| High | 2.531*** [1.527,4.195] | 1.995***[1.468,2.711] | 1.019 [0.638,1.627] | 0.698 [0.303,1.609] |
| Highest | 4.621*** [2.243,9.519] | 3.721***[2.582,5.362] | 1.028 [0.627,1.688] | 2.159 [0.565,8.260] |
| ANC (ref <4ANC) | 1.000 | 1.000 | 1.000 | 1.000 |
| 4+ANC | 2.527*** [1.891,3.377] | 2.516***[1.962,3.226] | 2.689*** [1.986,3.640] | 2.311**[1.271,4.202] |
| Treatment (Ref, Comparison) | 1.000 | 1.000 | 1.000 | 1.000 |
| Intervention | 11.880*** [6.391,22.083] | 0.373***[0.305,0.458] | 0.98 [0.718,1.337] | 0.788 [0.424,1.463] |
| Constant | 2.265 [0.515,9.968] | 0.845 [0.541,1.320] | 3.591** [1.414,9.118] | 13.220***[3.423,51.056] |
| Total N | 2,937 | 1,941 | 2,274 | 856 |
|  |  |  |  |  |
| ***PNC*** |  |  |  |  |
| Marital status (ref, Single/divorced/widowed) | 1.000 | 1.000 | 1.000 | 1.000 |
| Married | 0.666 [0.270,1.638] | 1.172 [0.946,1.452] | 1.088 [0.820,1.445] | 1.158 [0.784,1.710] |
| Mother’s Education (ref, none) | 1.000 | 1.000 | 1.000 | 1.000 |
| Primary | 1.244 [0.955,1.622] | 1.041 [0.815,1.329] | 0.897 [0.520,1.548] | 2.991** [1.408,6.353] |
| Secondary or more | 1.274 [0.933,1.738] | 1.093 [0.756,1.582] | 1.269 [0.723,2.228] | 3.284** [1.517,7.108] |
| Mother’s age (ref, <20y) | 1.000 | 1.000 | 1.000 | 1.000 |
| ≥20y | 1.382 [0.846,2.258] | 1.106 [0.757,1.616] | 1.198 [0.846,1.698] | 0.646 [0.399,1.044] |
| Parity (ref, 2 or more) | 1.000 |  | 1.000 | 1.000 |
| 1^st^ pregnancy | 0.867 [0.686,1.095] |  | 1.206 [0.793,1.834] | 1.062 [0.607,1.857] |
| Health insurance (ref, none) | 1.000 |  |  |  |
| Health insurance | 1.275 [0.998,1.628] |  |  |  |
| Wealth Quintile (ref, lowest) | 1.000 | 1.000 | 1.000 | 1.000 |
| Low | 1.315 [0.986,1.753] | 1.282 [0.943,1.744] | 1.2 [0.927,1.553] | 1.693 [0.962,2.980] |
| Middle | 1.38 [0.990,1.923] | 1.278 [0.925,1.768] | 1.141 [0.806,1.616] | 3.127*** [1.858,5.261] |
| High | 1.442* [1.011,2.057] | 0.994 [0.718,1.377] | 1.293 [0.980,1.706] | 5.913*** [3.480,10.046] |
| Highest | 1.323 [0.903,1.937] | 1.389 [0.971,1.988] | 1.145 [0.866,1.515] | 8.352*** [4.454,15.660] |
| ANC (ref <4ANC) | 1.000 | 1.000 | 1.000 | 1.000 |
| 4+ANC | 1.202 [0.958,1.508] | 1.034 [0.809,1.321] | 1.509*** [1.249,1.823] | 1.06 [0.752,1.494] |
| SBA (ref, no SBA) | 1.000 | 1.000 | 1.000 | 1.000 |
| SBA | 5.029*** [3.772,6.704] | 3.265***[2.616,4.076] | 5.444*** [3.352,8.842] | 2.121 [0.960,4.690] |
| Treatment (Ref, Comparison) | 1.000 | 1.000 | 1.000 | 1.000 |
| Intervention | 2.678*** [2.037,3.522] | 0.183***[0.148,0.227] | 1.145 [0.950,1.379] | 11.630***[7.702,17.560] |
| Constant | 0.64 [0.220,1.863] | 0.719 [0.450,1.149] | 0.058*** [0.026,0.129] | 0.008*** [0.002,0.028] |
| Total N | 2,936 | 1,940 | 2,267 | 851 |

*p<0.05, **p<0.01, ***p<0.001
